# Effects of Serial Passage on the Characteristics and Cardiac and Neural Differentiation of Human Umbilical Cord Wharton's Jelly-Derived Mesenchymal Stem Cells

**DOI:** 10.1155/2016/9291013

**Published:** 2015-12-20

**Authors:** Jianchun Lian, Shijie Lv, Chang Liu, Yang Liu, Shujun Wang, Xin Guo, Feng Nan, Hua Yu, Xin He, Guangwei Sun, Xiaojun Ma

**Affiliations:** ^1^Laboratory of Biotechnology, Dalian Institute of Chemical Physics, Chinese Academy of Sciences, Dalian 116023, China; ^2^The Laboratory Medical College, Dalian Medical University, Dalian 116044, China; ^3^Dalian Maternity & Child Healthcare Hospital, Dalian 116033, China; ^4^University of Chinese Academy of Sciences, Beijing 100049, China; ^5^Department of Orthopedics, The Second Hospital of Dalian Medical University, Dalian 116023, China

## Abstract

*Background and Objective*. It is important to guarantee the quality of stem cells. Serial passage is the main approach to expand stem cells. This study evaluated effects of serial passage on the biological characteristics of human umbilical cord Wharton's jelly-derived MSCs (WJ MSCs). *Methods*. Biological properties of WJ MSCs in the early (less than 10 passages, P10), middle (P11–20), and late (more than P20) phases including cell proliferation, cell cycle, phenotype, senescence, oncogene expression, stemness marker expression, and differentiation capacity were evaluated using flow cytometry, real-time PCR, immunocytofluorescence, and western blot. *Results*. It was found that there were no significant differences in cell proliferation, cell cycle, phenotype, and stemness marker expression in different phases. However, the expression of senescence-related gene, p21, and oncogene, c-Myc, was significantly upregulated in the late phase, which had close relations with the obviously increased cell senescence. Moreover, cardiac differentiation capability of WJ MSCs decreased whereas the propensity for neural differentiation increased significantly in the middle phase. *Conclusions*. This study reveals that WJ MSCs in the early and middle phases are relatively stable, and effect of serial passage on the lineage-specific differentiation should be considered carefully.

## 1. Introduction

Human mesenchymal stem cells (MSCs) have great potential in regenerative medicine, because they can self-renew and differentiate into cellular derivations of three primary germ layers, such as neural cells [1], cardiomyocytes [2], and hepatocytes [3]. MSCs reside in many adult organs or tissues, for example, bone marrow (BM) and adipose, and they also exist in birth-associated tissues, such as umbilical cord (UC), placenta, and amniotic fluid [4]. MSCs were isolated initially from BM, but BM aspiration is a highly invasive procedure, which is detrimental for patients [5]. Adipose tissue is thought to be a better source than BM because it can be obtained by a less invasive procedure [6]. However, it is reported that the quality of MSCs derived from BM or adipose tissue may decline with progressive age [7, 8]. Birth-associated tissues were discarded as medical waste before. However, at present, they are gaining popularity as alternative sources of MSCs [9, 10]. In birth-associated tissues accessed, UC Wharton's jelly-derived MSCs (WJ MSCs) offer the best clinical utility, partly due to their high purity and unique properties [11]. It is reported that WJ MSCs have higher expression of undifferentiated human embryonic stem cell (ESC) markers than BM MSCs, and they also can be induced to be neural progenitors with higher efficiency compared to BM MSCs and adipose-derived MSCs (AD MSCs) [12, 13]. In addition, more and more WJ MSCs are preserved in stem cell banks, which indicates that WJ MSCs have the potential for large-scale applications. Therefore, it appears that WJ MSCs may be an ideal MSC source instead of BM and AD MSCs.

To ensure the accuracy and stability of stem cell research, it is very important to guarantee the quality of cultured stem cells due to the possibility of lost phenotype, malignant transformation, altered lineage-specific differentiation capacity, and so forth during MSC culture in vitro. Serial passage is the main approach to culture MSCs in vitro at present. It has been found that serial passage has different effects on the cellular characteristics of human BM MSCs and AD MSCs [14–16]. However, how serial passage affects the biological characteristics of WJ MSCs is still unclear at present.

Therefore, in this study we investigated effects of serial passage on the specific characteristics of WJ MSCs in the early phase (less than 10 passages, representative passage 7, P7), middle phase (between 10 and 20 passages, P14), and late phase (more than 20 passages, P21) by concurrently monitoring cell proliferation, cell cycle, phenotype, senescence, oncogene expression, stemness marker expression, differentiation capacity, and so forth. Our results show that there are no significant differences in cell proliferation, cell cycle, phenotype, and stemness marker expression in different phases. However, the expression of senescence-related gene, p21, and oncogene, c-Myc, is significantly upregulated in the late phase, which has close relations with the obviously increased senescence of WJ MSCs. Moreover, cardiac differentiation capability of WJ MSCs might decrease whereas the propensity for neural differentiation might increase significantly in the middle phase. These results indicated that WJ MSCs in the early and middle phases were relatively stable, and the effect of serial passage on the lineage-specific differentiation of WJ MSCs would be considered carefully in stem cell research, which was important for the quality control of stem cells.

## 2. Material and Methods

### 2.1. WJ MSC Culture

Primary WJ MSCs from three human UC samples (*n* = 3) were isolated as previously described [12] and then donated by Zhongyuan Union Stem Cell Bioengineering Corporation (Tianjin, China). The cells were cultured in the special WJ MSC culture medium without animal serum that was also kindly gifted by Zhongyuan Union Stem Cell Bioengineering Corporation. The medium was changed every three days and cells were subcultured when reaching 90% confluence with the seeding density of 10^4^ cells/cm^2^. The morphology of WJ MSCs was observed under a microscope (Eclipse TE2000-U, Nikon, Tokyo, Japan). Population doubling (PD) and population doubling time (PDT) of the cultured WJ MSCs were calculated according to the previous report [17].

### 2.2. WJ MSC Proliferation

Cell proliferation was determined by using the cell counting kit-8 (CCK8) (Dojindo Laboratories, Kumamoto, Japan) assay according to the manufacturer's instructions [18]. The absorbance at 450 nm with a reference wavelength at 630 nm was recorded using a microplate reader (Well Scan MK3, Labsystems, Dragon, Finland).

### 2.3. Cell Cycle Analysis

WJ MSCs were harvested and fixed in 70% cold ethanol overnight. Cell pellets were resuspended in 1 mg/mL RNase (Sigma-Aldrich, St. Louis, MO, USA) at 37°C for 30 min, followed by staining with 50 *μ*g/mL propidium iodide (Sigma-Aldrich). For each sample, 10^4^ cells were analyzed by flow cytometry (FACSCalibur, BD, Berkeley, CA, USA). Results were expressed as the percentage of cells in each phase of the cell cycle.

### 2.4. Apoptosis Analysis

WJ MSCs were treated by using Annexin V-FITC/PI kit (KeyGEN bioTECH, Nanjing, China) according to the manufacturer's instruction. The stained cells were detected with the BD FACSCalibur [19].

### 2.5. Cell Senescence Analysis

SA-*β*-gal activity was detected by the SA-*β*-gal staining kit (Biyuntian, Wuhan, China) according to the manufacture's instruction [20]. The percentage of senescent cells was calculated according to 3 staining photographs selected at random in each experiment group.

### 2.6. Chromosome Analysis

The protocol was based on the previous report with some modifications [21]. In brief, when WJ MSCs (P7, P14, and P21) reached 70–80% confluence, culture medium was switched to fresh medium containing 0.15 *μ*g/mL colchamine (Sigma-Aldrich) for 3 h. Then cells were dissociated by trypsin-EDTA (Sigma-Aldrich). And cells were treated with the hypotonic solution (0.75 M KCl) (Sigma-Aldrich) at 37°C for 24 min. After that, cells were fixed for 20 min by using fresh Carnoy's fixative solution, 3 : 1 (v/v) methanol/glacial acetic acid (Sigma-Aldrich). Then cells were dropped from high altitude, air dried, and stained by Giemsa stain (Sigma-Aldrich) for 8 min. Samples were analyzed by chromosome analysis software.

### 2.7. Flow Cytometric Analysis

WJ MSCs were stained directly with FITC or PE-immunolabeled mouse anti-human monoclonal antibodies against HLA-DR, CD45, CD73, CD90, and CD105 (BD Pharmingen, San Diego, CA, USA). Analysis was performed with the FACSCalibur (BD). Data were expressed as number of cells/10^6^ cytometric events.

### 2.8. Cardiac Differentiation

Cardiac differentiation was performed according to the previous report [22]. Briefly, WJ MSCs were treated with 10 *μ*M 5-Aza (Sigma-Aldrich) for 24 h. After that, fresh medium was added for 14 days. Medium was changed every three days and the morphology of differentiated WJ MSCs was observed by using the microscope (Eclipse TE2000-U).

### 2.9. Neural Differentiation

Neural differentiation of WJ MSCs was performed according to the previous report with some modifications [23]. WJ MSCs were treated with 10 ng/mL bFGF for 24 h. After that, 1% (v/v) DMSO and 100 *μ*M *β*-hydroxyanisole (BHA, Sigma-Aldrich) were added for another 24 h. The morphology of differentiated WJ MSCs was also observed.

### 2.10. Real-Time Polymerase Chain Reaction

Total RNA was extracted using TRIzol reagent (TaKaRa, Shiga, Japan) according to manufacturer's protocol. Reverse transcription was performed with the PrimeScript RT reagent Kit (TaKaRa). PCR amplifications were performed using 2x SYBR Premix Ex Taq II (TaKaRa). The amplified signals were detected continuously with the Stratagene Mx3000P Real-Time Cycler (Agilent Technology, Santa Clara, USA). Primers (listed in Table 1) used in this study were designed by Takara Biotechnology (Dalian, China).

### 2.11. Immunocytofluorescence

After neural differentiation, cells were fixed in 4% paraformaldehyde (PFA, Sigma-Aldrich). Incubation with primary and secondary antibodies was carried out in PBS. For negative controls, the primary antibody was omitted. Nuclear staining was performed with Hoechst 33342 (Sigma-Aldrich). The sources of the antibodies used in this study included the following: anti-Nestin (Sigma-Aldrich), anti-*β*-Tubulin III (Sigma-Aldrich), and Alexa Fluor 488 goat anti-rabbit IgG antibody (Invitrogen, Carlsbad, CA, USA). The specimens were viewed with the fluorescence microscope (Eclipse TE2000-U). The percentage of Nestin or *β*-Tubulin III positive cells was calculated according to 3 immunostaining photographs selected at random in each experiment group (total cell number more than 300).

### 2.12. Western Blot

Cells were lysed in lysis buffer according to previous report [24] and were centrifuged at 12,000 g for 20 min, at 4°C. For each sample, 20 *μ*g total protein was electrophoresed through a 10% (w/v) acrylamide gel and then transferred to a nitrocellulose membrane (Bio-Rad Laboratories, Hercules, CA, USA). The blots were incubated at 4°C overnight with anti-*α*-actinin antibody (Sigma-Aldrich), and the resulting bands were detected by using diaminobenzidine- (DAB-) based horseradish peroxidase (HRP) reaction. Intensities of the bands were semiquantified using a Gel Logic 2200 PRO imaging system (Carestream Molecular Imaging, New Haven, Connecticut, USA).

### 2.13. Statistical Analysis

WJ MSCs from three human UC samples were used in this study (*n* = 3). All individual experiments were performed in triplicate. Data were expressed as means ± standard deviation (SD). One-way ANOVA was performed to test the significance of data. Differences were considered significant at ^*∗*^ *P* < 0.05 and especially significant at ^*∗∗*^ *P* < 0.01.

## 3. Results

### 3.1. Morphology, Proliferation, Cell Cycle, Apoptosis, and Senescence of WJ MSCs during Serial Passage

**Figure 1:**
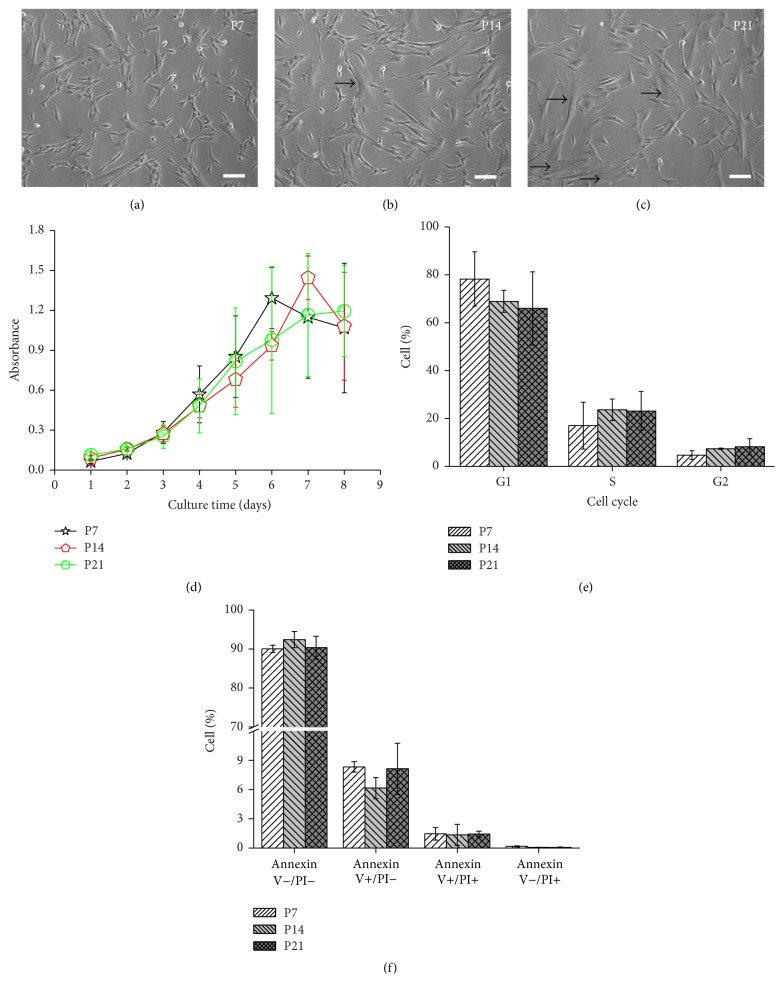
Morphology, proliferation, cell cycle, and apoptosis of WJ MSCs (Wharton's jelly-derived mesenchymal stem cells) in the early, middle, and late phases. (a) Representative phase-contrast micrographs showed that there were many small raised cells with a fibroblast-like appearance in the early phase, (b) then some cells gained an irregular and flat morphology in the middle phase, and (c) more cells appeared to be irregular and flat in the late phase. Arrows indicated the irregular and flat cells. Bar: 100 *μ*m. (d) CCK8 (cell counting kit-8) assay showed that the proliferation rate of WJ MSCs decreased gradually in long-term culture in vitro. (e) S and G2 phase block phenomenon might exist at higher passages (P14 and P21) compared to P7. (f) There were no significant differences in apoptosis among three phases.

It was found that the morphology of WJ MSCs changed during serial passage. WJ MSCs contained many small raised cells with a fibroblast-like appearance in the early phase (Figure 1(a), P7) while becoming larger and more elongate, and some cells gained an irregular and flat morphology in the middle phase (Figure 1(b), P14). In the late phase, more cells appeared to be irregular and flat, and more inclusions were also found in the cytoplasm (Figure 1(c), P21). In addition, the mean PD of WJ MSCs in the early, middle, and late phases (P7, P14, and P21) was 3, 2.8, and 1.8, and the mean PDT was 24, 34.3, and 66.7 hours, respectively.

CCK8 assay showed that the proliferation rate of WJ MSCs decreased gradually in long-term culture in vitro (Figure 1(d)). Compared to cells in the late phase (P21), there was not a plateau in the growth curve of WJ MSCs in the early and middle phases (P7 and P14) (Figure 1(d)). It was probably due to the very high proliferative ability of P7 and P14 WJ MSCs, which resulted in too high cell density and the rapid decrease of the absorbance after reaching the peak value.

There were no significant differences in cell cycle among all the phases (Figure 1(e)). It was also found that S and G2 phase block phenomenon might exist at higher passages (P14 and P21) compared to P7.

In addition, there were no significant differences in apoptosis among three phases (Figure 1(f)). At different passages, the cell percentages of Annexin V−/PI− and Annexin V+/PI− were about 90% and 9%, respectively, which indicated serial passage did not lead to the significant apoptosis of WJ MSCs.

**Figure 2:**
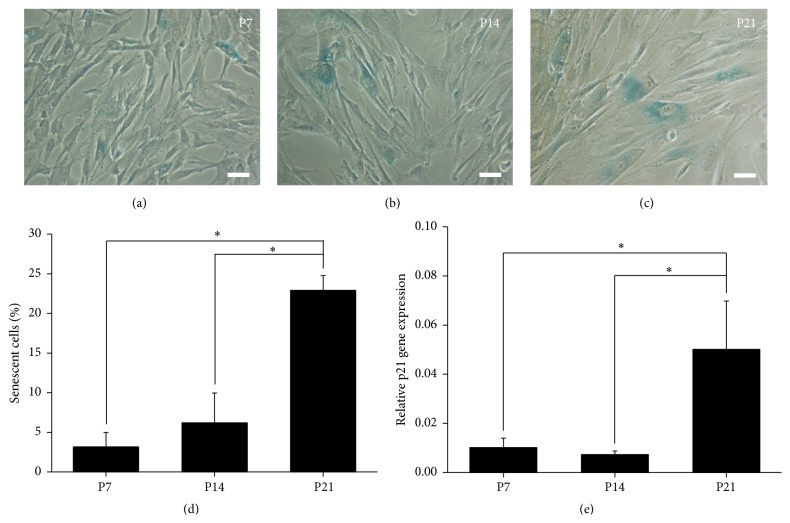
SA-*β*-gal staining, percentage of senescent cells, and p21 gene expression in WJ MSCs (Wharton's jelly-derived mesenchymal stem cells) in the early, middle, and late phases. (a) SA-*β*-gal staining showed that there were few senescent cells in WJ MSCs in the early phase, (b) senescent cells were found in the middle phase, and (c) more senescent cells appeared in the late phase. Bar: 100 *μ*m. (d) The percentage of senescent cells in WJ MSCs in the late phase was 22.9 ± 1.9%, significantly higher than 3.2 ± 1.8% in the early phase and 6.2 ± 3.8% in the middle phase (^*∗*^ *P* < 0.05). (e) The gene expression of p21 was upregulated in WJ MSCs in the late phase, which was also significantly higher than that in the early and middle phases (^*∗*^ *P* < 0.05).

SA-*β*-gal staining showed that there were few senescent cells in WJ MSCs in the early phase (Figure 2(a), P7). With serial passage, senescent cells were found in the middle phase (Figure 2(b), P14), while more senescent cells appeared in the late phase (Figure 2(c), P21). Statistical analysis results of SA-*β*-gal staining showed that the percentage of senescent cells in P21 WJ MSCs was 22.9 ± 1.9%, significantly higher than P7 (3.2 ± 1.8%) and P14 (6.2 ± 3.8%) (^*∗*^ *P* < 0.05) (Figure 2(d)). In addition, the profile of p21 gene expression, one of the senescence-related genes [20], was similar to the statistical analysis results of SA-*β*-gal staining. The gene expression of p21 was upregulated in WJ MSCs in the late phase, which was also significantly higher than that in the early and middle phases (^*∗*^ *P* < 0.05) (Figure 2(e)). The above results indicated that serial passage resulted in the senescence of WJ MSCs in the late phase significantly, which might be related to the p21 gene expression.

### 3.2. Oncogene Expression in WJ MSCs during Serial Passage

**Figure 3:**
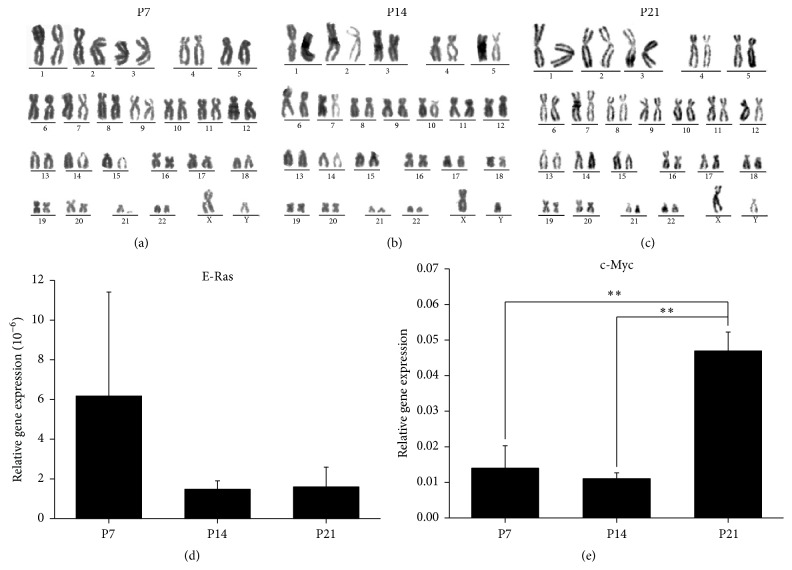
Chromosome analysis and the expression of oncogenes, E-Ras and c-Myc, in WJ MSCs (Wharton's jelly-derived mesenchymal stem cells) in the early, middle, and late phases. (a–c) Chromosome analysis indicated that serial passage did not change the number of chromosome in WJ MSCs regardless of the phases. (d) There were no significant differences in E-Ras gene expression among different phases. (e) The c-Myc gene expression in WJ MSCs in the late phase was upregulated, remarkably higher than the early phase and the middle phase (^*∗∗*^ *P* < 0.01).

Chromosome analysis indicated that serial passage did not change the number of chromosome in WJ MSCs regardless of the phases (Figures 3(a)–3(c)). E-Ras and c-Myc are oncogenes. It was found that there were no significant differences in E-Ras gene expression among different phases, but large individual difference was found in P7 WJ MSCs (Figure 3(d)). However, the c-Myc gene expression in WJ MSCs in the late phase (P21) was upregulated, significantly higher than that in the early phase (P7) and the middle phase (P14) (^*∗∗*^ *P* < 0.01) (Figure 3(e)).

### 3.3. Surface Marker and Pluripotency Gene Expression in WJ MSCs during Serial Passage

It was found that WJ MSCs expressed classic MSC surface markers, including CD73, CD90, and CD105 at high level (about 99%), and they were negative for hematopoietic markers, CD45 and HLA-DR (Table 2). Moreover, there were no differences in cell phenotype among different phases, which indicated that serial passage had no significant effect on the classic surface markers of MSCs.

Although surface marker expression was stable during serial passage, the percentage of senescent cells and c-Myc gene expression was significantly increased in WJ MSCs in the late phase compared to the early and middle phases. This indicated that WJ MSCs in the early and middle phases might be more stable than cells in the late phase. Therefore, next, we investigated the pluripotency gene expression and committed differentiation of WJ MSCs in the early and middle phases.

**Figure 4:**
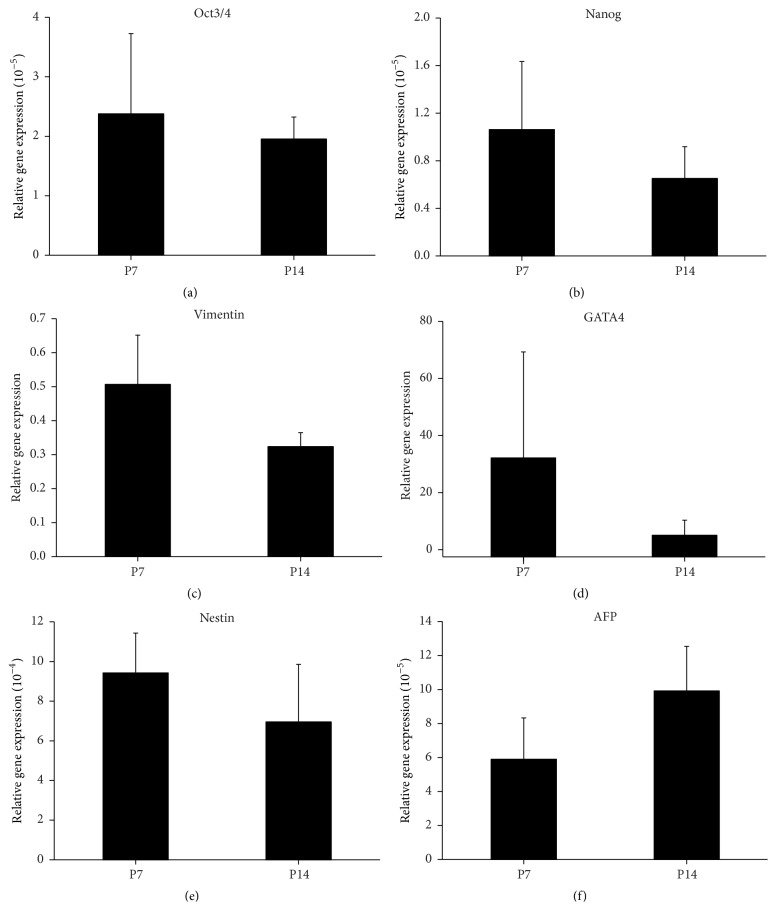
The expression of pluripotency genes and triploblastic progenitor marker genes in WJ MSCs (Wharton's jelly-derived mesenchymal stem cells) in the early and middle phases. (a–c) There was a downward trend in the expression of Oct3/4, Nanog, and Vimentin in the middle phase compared to the early phase. (d–f) The expression of GATA4, Nestin, and AFP in the early and middle phases.

WJ MSCs expressed ESC marker genes, Oct3/4 and Nanog, and MSC marker gene, Vimentin. No significant differences in the expression of these genes between P7 and P14 were found, although there was a downward trend (Figures 4(a), 4(b), and 4(c)).

In addition, WJ MSCs also expressed triploblastic progenitor marker genes, for example, mesoderm GATA4, ectoderm Nestin, and entoderm AFP. It was found that there were no significant differences in the expression of these genes between P7 and P14 (Figures 4(d), 4(e), and 4(f)).

### 3.4. Cardiac Differentiation Capability of WJ MSCs during Serial Passage

**Figure 5:**
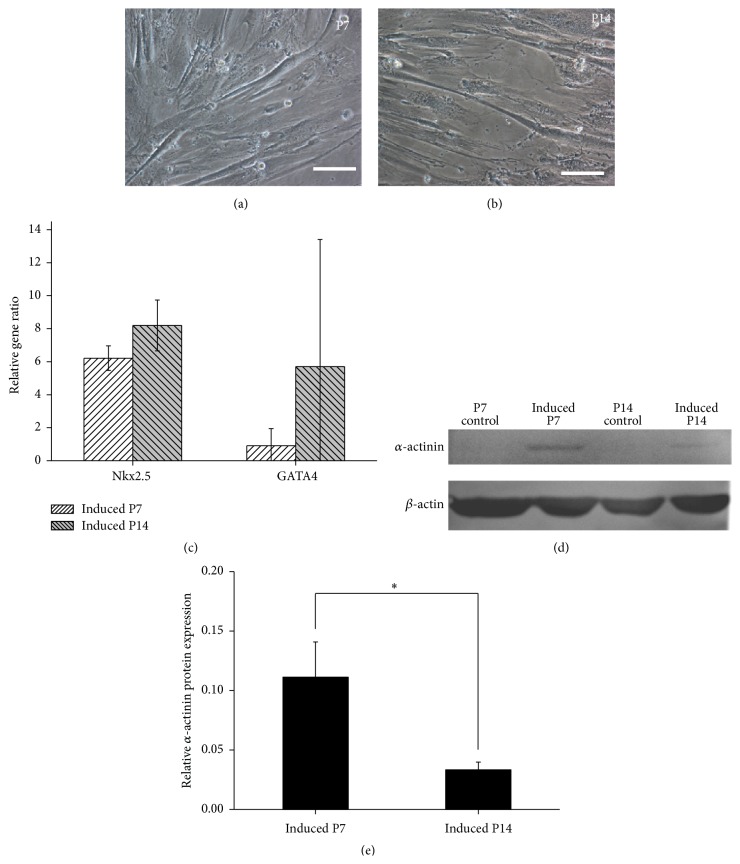
Morphology and expression of cardiac-specific genes and protein in WJ MSCs (Wharton's jelly-derived mesenchymal stem cells) in the early and middle phases after cardiac induction. (a-b) After being treated with 5-Aza for 14 days, cardiomyocyte-like cells with a stick-like morphology were found in induced P7 and P14 cells. Bar: 100 *μ*m. (c) The gene expression of GATA4 and Nkx2.5 was upregulated after cardiac induction. (d) No *α*-actinin protein expression was detected in normal P7 and P14 WJ MSCs (P7 control and P14 control). However, *α*-actinin protein expression appeared in the induced cells (induced P7 and induced P14). (e) Semiquantified analysis of western blot results showed that *α*-actinin protein expression in the group of induced P7 was significantly higher than induced P14 (^*∗*^ *P* < 0.05), which indicated that serial passage significantly decreased the cardiac differentiation capability of WJ MSCs.

WJ MSCs in the early (P7) and middle (P14) phases were treated with 5-Aza for 24 h to initiate cardiac differentiation. 14 days later, cardiomyocyte-like cells with a stick-like morphology were found in both two groups (induced P7, Figure 5(a)) (induced P14, Figure 5(b)).

The expression of GATA4 and cardiac-specific gene, Nkx2.5, was upregulated after cardiac induction (Figure 5(c)). The expression of both two genes was slightly higher in the group of induced P14 than induced P7 (Figure 5(c)). However, there were no significant differences in gene expression between these two groups.

Moreover, the expression of cardiomyocyte-related protein, *α*-actinin, was also detected by western blot. No *α*-actinin protein expression was detected in P7 and P14 WJ MSCs without induction (P7 control and P14 control) (Figure 5(d)). However, *α*-actinin protein expression was detected in the induced cells (Figure 5(d)). Semiquantified analysis results showed that *α*-actinin protein expression in the group of induced P7 was significantly higher than induced P14 (^*∗*^ *P* < 0.05) (Figure 5(e)). Therefore, serial passage significantly decreased the cardiac differentiation capability of WJ MSCs according to protein analysis results, although gene expression had an opposite trend.

### 3.5. Neural Differentiation Capability of WJ MSCs during Serial Passage

**Figure 6:**
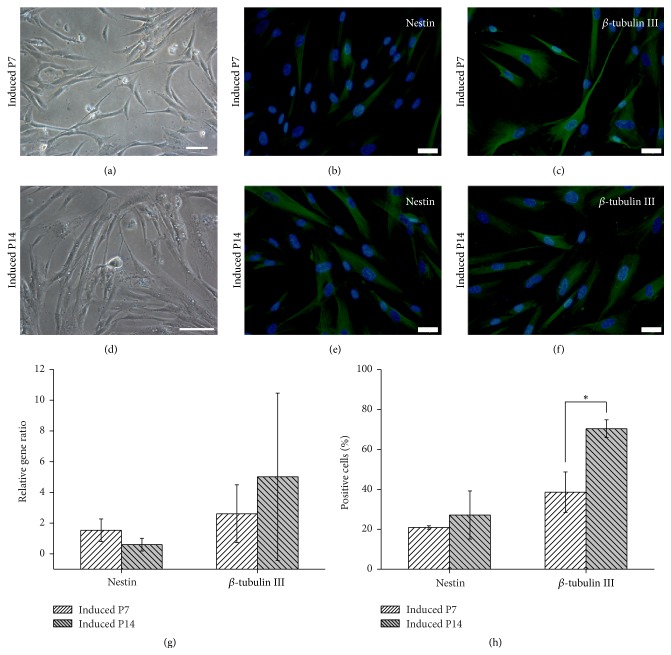
Morphology and expression of neural-specific genes and protein in WJ MSCs (Wharton's jelly-derived mesenchymal stem cells) in the early and middle phases after neural induction. (a and d) After DMSO and BHA (*β*-hydroxyanisole) treatment, WJ MSCs in the early and middle phases exhibited a neuronal-like morphology. Bar: 100 *μ*m. (b, c, e, f) Immunocytofluorescence staining results showed that neural induction significantly increased the protein expression of Nestin and *β*-Tubulin III in induced cells (induced P7 and induced P14). Bar: 50 *μ*m. (g) The gene expression of Nestin and *β*-Tubulin III was upregulated after neural induction with no significant differences between two groups (induced P7 and induced P14). (h) The percentage of *β*-Tubulin III positive cells in the group of P14 induction (70.4 ± 4.5%) was significantly higher than P7 induction (38.6 ± 10.2%) according to the statistical analysis results (^*∗*^ *P* < 0.05), which indicated that serial passage significantly increased the neural differentiation capability of WJ MSCs.

After DMSO and BHA treatment, both WJ MSCs in the early (P7) and middle (P14) phases exhibited a different morphology (induced P7, Figure 6(a)) (induced P14, Figure 6(d)).

The gene expression of neural progenitor marker, Nestin, and neuronal marker, *β*-Tubulin III, was upregulated after neural induction with no significant differences between these two groups (induced P7 and induced P14) (Figure 6(g)). And big individual differences in *β*-Tubulin III gene expression were found in the group of induced P14 compared to induced P7.

Except gene expression, we also investigated the protein expression of Nestin and *β*-Tubulin III in both two groups by immunocytofluorescence staining. Although few WJ MSCs were positive for either Nestin or *β*-Tubulin III in the early (P7) and middle (P14) phases (data not shown), neural induction significantly increased the protein expression of these two markers in both two groups (induced P7, Figures 6(b) and 6(c)) (induced P14, Figures 6(e) and 6(f)). Moreover, it was found that the percentage of *β*-Tubulin III positive cells in the group of P14 induction (70.4 ± 4.5%) was significantly higher than P7 induction (38.6 ± 10.2%) according to the statistical analysis results (^*∗*^ *P* < 0.05) (Figure 6(h)), while no significant differences were found in the percentage of Nestin positive cells between both groups (induced P7, 20.9 ± 0.8%, and induced P14, 27.2 ± 12%). Therefore, these results indicated that serial passage significantly increased the neural differentiation capability of WJ MSCs.

## 4. Discussion

During serial passage, we found that the senescence of WJ MSCs was increased significantly in the late phase (22.9 ± 1.9%), compared to the middle phase (6.2 ± 3.8%) and the early phase (3.2 ± 1.8%) (^*∗*^ *P* < 0.05) (Figure 2(d)). Cheng et al. investigated the senescence of whole UC tissue-derived MSCs in the early phase (P2–10), the middle phase (P11–20), and the late phase (more than P20), and they found that the percentage of senescent cells was increased significantly in the late phase (76%), compared to the middle phase (28%) and the early phase (very few) [20], which had the similar trend to our results. However, the percentage of senescent cells either in the middle (P14) or late phase (P21) was much lower in this study compared to their results. On one hand, the difference in cell properties between WJ MSCs and whole UC tissue-derived MSCs might lead to this distinction. Except Wharton's jelly, whole UC tissue also contains other compartments, including amnion, subamnion, perivascular region, and umbilical blood vessel endothelium [11]. Several types of stem cell populations with varied stemness properties exist in these compartments [25]. So whole UC tissue-derived MSCs contain mixed stem cell populations with varied properties [11], which may result in the difference in senescence between WJ and whole UC tissue-derived MSCs. On the other hand, MSC culture medium might also have relations with the senescence. Cheng et al. used DF-12 medium containing 10% FBS while WJ MSCs were cultured in special culture medium without animal serum gifted by a company in this study. This special culture medium is developed for stem cell therapy in the future. Although the recipe of the special culture medium was unknown, it seemed that this optimized culture medium is significantly beneficial to reduce the senescence of WJ MSCs during long-term culture.

It is reported that p21 gene, one of the senescent-related genes, is important for whole UC tissue-derived MSCs [20, 26]. And the increased c-Myc gene expression in AD MSCs is also relevant to the senescence during serial passage [27]. In this work, we found that both p21 and c-Myc gene expression in WJ MSCs were increased significantly in the late phase (^*∗*^ *P* < 0.05, Figure 2(e)) (^*∗∗*^ *P* < 0.01, Figure 3(e)), which showed the same trend as the percentage of senescent cells (^*∗*^ *P* < 0.05, Figure 2(d)). Therefore, our results indicated that both p21 and c-Myc gene expression had close relations with the senescence of WJ MSCs during long-term culture in vitro, and these two genes may be used as markers for senescence monitoring.

Except the senescence, the upregulated c-Myc gene expression is also associated with in vitro spontaneous transformation of human MSCs [27]. Sawada et al. found that the c-Myc gene expression in human MSCs from some donors could be significantly upregulated in long-term culture, accompanied with the increased percentage of c-Myc aberrant cells [28], which indicated that long-term culture might increase the safety risk of MSCs and c-Myc expression was useful for the early evaluation. In this study, we found that c-Myc gene expression was significantly increased in WJ MSCs in the late phase (^*∗∗*^ *P* < 0.01, Figure 3(e)), which showed that the safety risk of WJ MSCs might be increased significantly in the late phase. Next, it is well worth evaluating the in vivo tumorigenicity of WJ MSCs with higher c-Myc gene expression after serial passage.

In this study, the cardiac differentiation capability of WJ MSCs decreased (^*∗*^ *P* < 0.05) (Figure 5(e)) whereas neural differentiation capability increased significantly (^*∗*^ *P* < 0.05) (Figure 6(h)) in the middle phase compared to the early phase. In addition, for whole UC tissue-derived MSCs, adipogenic differentiation decreases while osteogenic differentiation starts to dominate during serial passage [20]. These results indicated that the effect of serial passage might be relevant to the lineage-specific differentiation of WJ MSCs.

However, it was also found that the SD of the gene expression data, such as GATA4 (Figure 5(c)) and *β*-Tubulin III (Figure 6(g)), was larger between different UC samples after cardiac or neural induction, which was probably due to the individual difference. The properties of fetal stem cells are reported to have relations with pregnancy [29]. This indicates that, next, more UC samples should be investigated, and some clinical indexes, including volume and weight of UC or placenta of the puerpera, weight and age of the puerperal, and weight and development of the neonate, should be recorded carefully. By statistical analysis, we might find the possible relationship between stem cell properties and clinical indexes, which is very helpful to obtain better UC samples containing WJ MSCs with higher quality and stability. Although it was found that there were significant differences in *α*-actinin protein expression or *β*-Tubulin III positive cells after cardiac or neural differentiation between P7 and P14 in this study, however, it is necessary to check more markers and perform functional assays to further evaluate stem cell differentiation [30].

In addition, there are some different variation trends among MSCs from different sources during serial passage, such as surface marker expression and differentiation capacity. For AD MSCs, CD105 expression declines during serial passage [15]. However, for BM and WJ MSCs, the expression of CD105 and CD73 is maintained at high level during long-term culture [16, 31] (Table 2). Moreover, long-term culture does not decrease the neuronal differentiation capability of BM MSCs [16] while neural differentiation capability increased significantly in the middle phase compared to the early phase in this study (Figure 6(h)). These results indicate that effects of serial passage on MSC properties are also dependent on MSC sources.

## 5. Conclusion

We found that the expression of senescence-related gene, p21, and oncogene, c-Myc, was significantly upregulated in the late phase, which had close relations with the obviously increased senescence of WJ MSCs. And cardiac differentiation capability of WJ MSCs decreased whereas the propensity for neural differentiation increased significantly in the middle phase compared to the early phase. These results indicated that WJ MSCs in the early and middle phases were relatively stable, and the effect of serial passage on the lineage-specific differentiation of WJ MSCs would be considered carefully in stem cell research. Therefore, it is necessary to monitor the quality of WJ MSCs during culture in vitro, which would be helpful to choose relatively stable stem cells according to specific purposes, maximize the potential of stem cells, and improve the reproducibility of strategies and biotechnologies developed based on stem cells.

## Figures and Tables

**Table 1:** Real-time PCR primers used in the experiments.

Target	Forward primer	Reverse primer	Genbank acc. number
p21	5′-GGACAGCAGAGGAAGACCATGT-3′	5′-CGGCGTTTGGAGTGGTAGAA-3′	NM_000389
E-Ras	5′-GCAAGAGTGCGCTGACCAT-3′	5′-GCCCAGCACACCATCACA-3′	NM_181532.3
c-Myc	5′-CCACAGCAAACCTCCTCACA-3′	5′-CGGTTGTTGCTGATCTGTCTCA-3′	NM_002467
Oct3/4	5′-GTGGAGGAAGCTGACAACAATGAAA-3′	5′-GACCGAGGAGTACAGTGCAGTGAAG-3′	NM_002701
Nanog	5′-CAAAGGCAAACAACCCACTT-3′	5′-ATTGTTCCAGGTCTGGTTGC-3′	XM_002344636.1
Vimentin	5′-GGTGGACCAGCTAACCAACGA-3′	5′-TCAAGGTCAAGACGTGCCAGA-3′	NM_003380.3
Gata4	5′-GTTTTTTCCCCTTTGATTTTTGATC-3′	5′-AACGACGGCAACAACGATAAT-3′	NM_002052
Nestin	5′-GGGTCTACAGAGTCAGATCGCTCA-3′	5′-AGCGAGAGTTCTCAGCCTCCA-3′	NM_006617
AFP	5′-TAAACCCTGGTGTTGGCCAG-3′	5′-ATTTAAACTCCCAAAGCAGCAC-3′	NM_001134
Nkx2.5	5′-CCCCTGGATTTTGCATTCAC-3′	5′-CGTGCGCAAGAACAAACG-3′	NM_004387
*β*-Tubulin III	5′-CCTATTCAGGCCCGACAACTTTA-3′	5′-CAGGCAGTCACAATTCTCACACTC-3′	NM_006086
*β*-actin	5′-TGGCACCCAGCACAATGAA-3′	5′-CTAAGTCATAGTCCGCCTAGAAGCA-3′	NM_001101

**Table 2:** Surface marker analysis of WJ MSCs at different passages (*n* = 3).

Passage	HLA-DR	CD45	CD73	CD90	CD105
7	0.59 ± 0.54%	0.57 ± 0.45%	99.92 ± 0.07%	99.98 ± 0.03%	99.74 ± 0.13%
14	0.52 ± 0.15%	0.57 ± 0.11%	99.94 ± 0.04%	99.99 ± 0.01%	99.95 ± 0.05%
21	0.04 ± 0.06%	0.03 ± 0.04%	99.95 ± 0.03%	99.91 ± 0.1%	99.9 ± 0.09%
